# Is Congenital Amusia a Disconnection Syndrome? A Study Combining Tract- and Network-Based Analysis

**DOI:** 10.3389/fnhum.2017.00473

**Published:** 2017-09-29

**Authors:** Jieqiong Wang, Caicai Zhang, Shibiao Wan, Gang Peng

**Affiliations:** ^1^Department of Chinese and Bilingual Studies, The Hong Kong Polytechnic University, Hong Kong, China; ^2^Shenzhen Institutes of Advanced Technology, Chinese Academy of Sciences, Shenzhen, China; ^3^Department of Electrical Engineering, Princeton University, Princeton, NJ, United States

**Keywords:** congenital amusia, disconnection syndrome, tract-based spatial statistics, graph theory, white matter tract

## Abstract

Previous studies on congenital amusia mainly focused on the impaired fronto-temporal pathway. It is possible that neural pathways of amusia patients on a larger scale are affected. In this study, we investigated changes in structural connections by applying both tract-based and network-based analysis to DTI data of 12 subjects with congenital amusia and 20 demographic-matched normal controls. TBSS (tract-based spatial statistics) was used to detect microstructural changes. The results showed that amusics had higher diffusivity indices in the corpus callosum, the right inferior/superior longitudinal fasciculus, and the right inferior frontal-occipital fasciculus (IFOF). The axial diffusivity values of the right IFOF were negatively correlated with musical scores in the amusia group. Network-based analysis showed that the efficiency of the brain network was reduced in amusics. The impairments of WM tracts were also found to be correlated with reduced network efficiency in amusics. This suggests that impaired WM tracts may lead to the reduced network efficiency seen in amusics. Our findings suggest that congenital amusia is a disconnection syndrome.

## Introduction

Congenital amusia, also known as tone deafness, affects about 4% of the global population (Peretz and Hyde, 2003). Individuals with amusia are usually unable to distinguish between musical tones with small differences or recognize familiar melodies. This disorder is usually diagnosed based on the score on the Montreal Battery of Evaluation of Amusia (Peretz et al., 2003) or the Online Identification Test of Congenital Amusia (Peretz et al., 2008), which includes a series of tests that assess the subject’s ability to perceive musical pitch intervals, pitch contours, and rhythm. Although congenital amusia is easy to diagnose, its underlying cause is hard to explain, as it does not appear to be associated with visible brain lesions, hearing loss, or lack of environmental stimulation (Peretz et al., 2002). Behavioral studies link musical impairment to deficits in pitch perception (Foxton et al., 2004; Hyde and Peretz, 2004; Albouy et al., 2016) and pitch memory (Gosselin et al., 2009; Tillmann et al., 2009; Williamson et al., 2010; Albouy et al., 2016), which suggests potential changes in brain structures and/or brain functions. A full understanding of the neural bases of congenital amusia remains to be achieved.

Recently, techniques of non-invasive imaging have been applied to the investigation of brain abnormalities in amusics. Using T1-weighted imaging, researchers have found: (1) abnormalities in grey matter (GM) and white matter (WM) concentration in the right inferior frontal gyrus (IFG) and the right superior temporal gyrus (STG) of amusics via voxel-based morphology (Hyde et al., 2006; Mandell et al., 2007; Albouy et al., 2013); and (2) thicker cortices in the right IFG and the right auditory cortex via thickness analysis (Hyde et al., 2007). These findings suggest that the fronto-temporal pathway may be impaired in the brains of congenital amusics. The impaired fronto-temporal pathway in amusics is also consistent with the abnormal functional connectivity revealed by functional MRI (Hyde et al., 2011) and magnetoencephalography (Albouy et al., 2013).

Despite the plausibility of the fronto-temporal pathway hypothesis, it remains unclear whether the WM connections between the frontal and temporal regions are truly changed or not. So far, very few diffusion tensor imaging (DTI) studies have investigated the anatomical alterations of WM tracts in congenital amusia, and the results obtained are highly inconsistent (Loui et al., 2009; Chen et al., 2015). Loui et al. (2009) reported smaller fiber volume in amusics’ arcuate fasciculus (AF), the fiber connecting the frontal and temporal regions, which was identified by deterministic tractography (Basser et al., 2000), but this finding was criticized by Chen et al. (2015), who argued that the volume changes in AF found by Loui et al. (2009) may be a result of the specific tractography algorithm used. By contrast, Chen et al.’s (2015) study found no volume changes in the AF of amusics using deterministic and probabilistic tractography. Although Chen et al. (2015) suggested that probabilistic tractography performed better in detecting AF than deterministic tractography, they also acknowledged that the AF reconstructed by their method may not include the peripheral branches.

Thus the impairment of WM connections in amusics remains controversial, which requires further investigation. Furthermore, while previous studies focused exclusively on the AF, it is possible that WM tracts beyond the AF are also affected. A recent study applied the graph theory to provide a holistic prospective on anatomical topology (Zhao et al., 2016), which showed that the efficiency of WM network was reduced. Zhao et al. (2016) supposed that the reduced efficiency has the same trend with the structural abnormalities previously found in the fronto-temporal pathway (Hyde et al., 2006, 2011; Mandell et al., 2007). However, this study only focused on the changes in the whole network, and yet offered little understanding of the possible alterations in specific WM tracts. In the current study, we combined tract-based and network-based analysis to measure changes in WM tracts at both the local level and the global level. More importantly, we also examined the relationship between local and global differences in WM tracts.

The tract-based spatial statistics (TBSS) method and direct comparison of the strength of structural connections were applied in the local-level analysis of WM tracts. Graph theory was used in the global network-based analysis. It is likely that abnormal local WM tracts are associated with reduced efficiency of the brain network. In order to address this question, we also performed correlation analysis between the altered indices of tract-based analysis and the altered measurements of network-based analysis. The tract-based analysis and the network-based analysis are discussed below.

With DTI scans, several measurements can be obtained to assess the changes in WM tracts, such as fractional anisotropy (FA), mean diffusivity (MD), radial diffusivity (RD), and axial diffusivity (AD). Generally, these four measurements are sensitive to microstructural changes in WM, the microstructural architecture of cellular membranes, the myelin in WM, and axonal degeneration, respectively (Alexander et al., 2007). Although the direct comparison of measurements at each voxel across different groups can reveal local changes, the results of this method can be significantly and adversely affected by unavoidable registration errors and noise (Faria et al., 2010). In contrast, the TBSS method (Smith et al., 2006) can reduce these effects by projecting the volumetric data onto a WM skeleton. In this paper, we used TBSS to assess whether WM tracts are altered in amusics using these four measurements. In addition, we conducted a direct comparison of the number of WM tracts between pairs of structures to investigate whether the strength of structural connections is different in amusics.

The overall form of WM tracts can be assessed by characterizing the brain network using graph theory. WM tracts transfer information between different brain structures so that the whole brain can be seen as a network. Graph theory regards the brain network as a graph composed of a bunch of nodes (brain regions) and edges between nodes (WM tracts) (Rubinov and Sporns, 2010). A series of topological measurements obtained through graph theory were used to quantify the efficiency of information transfer and the functional integration/segregation of the brain, in terms of global efficiency (Latora and Marchiori, 2001), small-worldness (Humphries et al., 2006), modularity (Newman, 2006), and so on. To perform this network-based analysis, we constructed the WM tracts of the whole brain using deterministic tractography, and obtained the adjacent matrix of the brain network based on the WM tracts. Graph theory analysis was applied to investigate changes of topological measurements in congenital amusia.

In the present study, we hypothesize that congenital amusia is a disconnection syndrome with broad impairment of WM connections across the whole brain. Based on this hypothesis, we expect to find that in amusics (1) structural connections of the brain beyond the fronto-temporal pathway are altered, (2) topological network measurements are altered, (3) the altered indices of tract-based analysis are correlated with the altered topological measurements of network-based analysis, and (4) the altered measurements are correlated with musical scores.

## Materials and Methods

### Subjects

This study was approved by the Ethics Committee of the Chinese University of Hong Kong and the Institutional Review Board of the Shenzhen Institutes of Advanced Technology. Thirteen amusics (4 male, 9 female; age: 22 ± 3.42) and 23 age- and gender-matched normal controls (10 male, 13 female; age: 21.61 ± 2.87) were recruited and their written informed consent obtained. One participant was excluded from analysis because of history of epilepsy. All remaining participants were undergraduate students, right-handed native Cantonese speakers, with no reported history of brain injury or other psychiatric disorders, no long-term music training or hearing impairment.

Amusia diagnosis is based on the score on the Online Identification Test of Congenital Amusia (Peretz et al., 2008), which includes three subtests (i.e., out-of-key, offbeat, and mistune) to assess subjects’ musical pitch and rhythm perception (Peretz et al., 2002). The musical score is the average score of the score of out-of-key, the score of offbeat and the score of mistune. All amusics achieved an overall score (i.e., the average score of all three subtests) 73 or lower, and all controls a score of 80 or higher. The overall score was significantly lower among the amusics than the controls (*p* < 0.001).

### Data Acquisition

A Magnetom TRIO Scanner (Siemens, Erlangen, Germany) equipped with a 12-channel phased array receive-only head coil was used to obtain DTI data and T1-weighted imaging at the Shenzhen Institutes of Advanced Technology, the Chinese Academy of Sciences. All subjects were asked to keep their eyes closed but to remain awake in the scanner in a head-first supine position. The scanning parameters of the DTI data were as follows: TR/TE = 8300/88 ms, slice thickness = 2 mm, number of slices = 65, flip angle = 90°, acquisition matrix = 122 × 122, in-plane resolution = 2 × 2 mm^2^, and FOV = 244 × 244 mm^2^. The DTI scanning was first conducted without diffusion weighting (*b* = 0) and then the diffusion sensitizing gradients were applied along 30 non-collinear directions (*b* = 1000 s/mm^2^). The scanning process was subsequently repeated, totalling 62 volumes (two runs).

The scanning parameters of the T1-weighted images were: TR/TE = 2530/2.01 ms, slice thickness = 1 mm, number of slices = 192, flip angle = 7, matrix = 256 × 224, in-plane resolution = 1 × 1 mm^2^, and FOV = 256 × 224 mm^2^.

Two subjects, one normal control and one amusic, were discarded due to their T1-weighted images were missing. We also excluded one normal control subject because the DTI data were not scanned correctly. Finally, 20 normal controls and 12 amusics were used to perform further analysis. The demographic details of normal controls and amusics are shown in **Table 1**.

**Table 1:** The demographic details of normal controls and amusics.

		Normal controls (*n* = 20)	Amusics (*n* = 12)	*p*-value
Gender (male/female)		9/11	3/9	0.164
Age	Mean ± SD	21.75 ± 3.40	21.58 ± 3.00	0.890
	Range	18–33	18–28	n/a
Musical scores	Mean ± SD	89.55 ± 3.95	65.58 ± 5.43	<0.001
	Range	82–97	55–73	n/a

*Fisher’s exact test was used for gender match in two groups, while two-sample *t*-test was used for age and musical scores. Musical scores were obtained using the Online Identification Test of Congenital Amusia. SD, standard deviation; n/a, not applicable.*

### Preprocessing

FSL V5.0.9 software^1^ was used to preprocess the DTI data. First, eddy current distortion and simple head motion were corrected by registering all DTI volumes to the first b0 volume. The two corrected DTI runs were subsequently averaged to obtain an average run. Next, the FSL Brain Extraction Tool was applied to the average run with a fractional intensity threshold of 0.2 in order to obtain a binary brain mask for each subject. The average run and the corresponding brain mask were then used to fit a diffusion tensor model at each voxel using FDT and obtain the eigenvalues of the diffusion tensor matrix (λ1, λ2, λ3). The maps of fractional anisotropy (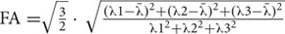, where 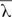 is the mean value of three eigenvalues), mean diffusivity [MD = (λ1+λ2+λ3)/3], radial diffusivity [RD = (λ2+λ3)/2], and axial diffusivity (AD = λ1) were generated based on these eigenvalues.

### Tract-Based Analysis

#### TBSS

The voxel-wise analysis of WM was conducted using TBSS (Smith et al., 2006). Instead of analyzing whole-brain voxels, standard TBSS mainly focuses on the voxels of a WM skeleton. First, FA images of all subjects were non-linearly registered to a standard-space image (FMRIB58_FA) provided by FSL. Then we calculated the mean map of all standard-space FA images, which was used to generate a skeleton image of WM tracts with the FA skeletonization program. An intensity threshold of 0.2 was used to define the boundary between WM and GM. The standard-space FA image of each subject was projected onto the skeleton in order to carry out the voxel-wise statistics across subjects. The same projection was also applied to the MD, RD, and AD images.

Voxel-wise group comparisons of the skeleton images were performed using the Randomize tool in FSL. This tool uses a non-parametric test with 10,000 permutations to identify group difference. The threshold of statistical significance was set at *p* < 0.001, corrected for multiple comparisons with the threshold-free cluster enhancement method (Smith and Nichols, 2009). The JHU–ICBM-DTI-81 WM tractography atlas and the Talairach Daemon atlas offered by FSL were used to identify the abnormal clusters revealed by TBSS. The JHU–ICBM-DTI-81 WM tractography atlas is probabilistic. The WM tract with the highest probability was chosen to label the abnormal cluster. The comparisons of MD, AD, and RD between normal controls and amusics were analyzed in the same way.

#### Strength of Structural Connections

Diffusion tensor imaging-Studio software (Jiang et al., 2006) was used to perform fiber tracking. First, the average run obtained in the preprocessing was used to calculate diffusion tensors and FA at each voxel using a linear least square fitting algorithm (Jiang et al., 2006). Then the fiber assignment was performed by a continuous deterministic tracking algorithm (Mori et al., 1999; Xue et al., 1999) to obtain WM fibers. In this process the tractography was computed by seeding each voxel with an FA over 0.2, and terminated when it reached a voxel with FA below 0.2 or it turned over 45°.

The commonly used MNI atlas, AAL atlas (Tzourio-Mazoyer et al., 2002), was used to parcellate the brain into 90 regions (45 in each hemisphere). In order to conduct parcellation in the native space, the AAL atlas was inversely transformed to the native space by transforming the T1-weighted imaging to MNI space. Then each subject’s brain was parcellated into 90 cortical or subcortical regions in the native space (Supplementary Table S1). In order to identify differences in connecting strength between the normal controls and amusics, the number of WM fibers between each two regions was compared between the two groups using a two-sample *t*-test. The significant statistical threshold was set at family-wise error (FWE) correction (*p* < 0.05).

### Network-Based Analysis

In graph theory, the brain is modeled as a graph in which each region parcellated by AAL atlas represents a node and the number of WM fibers between two regions represents the weight of the edge between two nodes. Here, a weighted WM network was constructed for each subject, represented as a symmetric 90 × 90 matrix (Supplementary Figure S1).

A wide range of thresholds from 1 to 10 was applied to threshold the weighted network. Then the following graph theoretical analysis was conducted on the thresholded network with different thresholds. The topological organization of the WM network was measured based on the symmetric 90 × 90 matrix, which was characterized by global topological properties. In the current study, the global properties included global efficiency (*Eg*) (Latora and Marchiori, 2001, 2003), mean local efficiency (*Eloc*) (Latora and Marchiori, 2001, 2003), modularity (*Mod*) (Newman, 2006), and small-worldness (σ) quantified by normalized clustering coefficient (γ) over normalized characteristic path length (λ) (Humphries et al., 2006). All the calculations of topological properties were based on the definitions offered by Rubinov and Sporns (2010) and were performed by MATLAB toolbox GRETNA V1.2.0^2^ (Wang et al., 2015). The topological properties in the current study are listed in Supplementary Table S2. For more details, please see Rubinov and Sporns’ recent review (Rubinov and Sporns, 2010).

The global properties of the two groups were compared using permutation-based non-parametric tests with 10,000 permutations at each threshold. False discovery rate (FDR) correction (*p* < 0.05) was performed to reduce the rate of Type I errors.

### Exploratory Research on Relationships between Local Changes and Global Changes

In order to investigate the relationship between the local changes identified by tract-based analysis and the global changes identified by network-based analysis, exploratory calculations of Pearson correlation coefficients between the altered indices of tract-based analysis and the altered topological measurements of network-based analysis were conducted in the control and amusia groups, respectively.

### Linking Changes with Music Performance

Pearson correlation coefficients between the altered measurements and scores on the Online Identification Test of Congenital Amusia were also calculated to investigate the relationship between altered measurements and amusia, in the control group and in the amusia group, respectively. We also correlated the altered brain measurements with the scores of three subtests, respectively. The significant threshold was set at 0.05 (FDR correction).

## Results

### Tract-Based Analysis

#### TBSS

**FIGURE 1:**
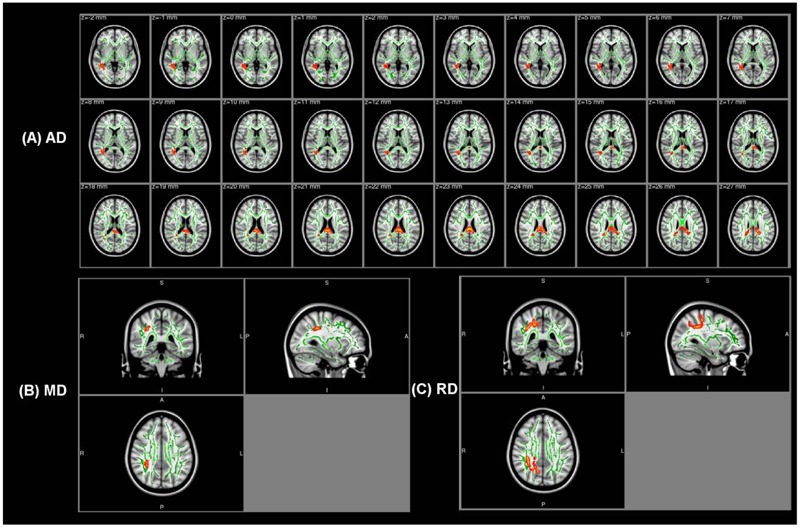
The white fiber tracts with higher AD/MD/RD values in the amusia group. **(A)** The white fiber tracts with higher AD values (*p* < 0.001, corrected for multiple comparisons). **(B)** The white fiber tracts with higher MD values (*p* < 0.001, corrected for multiple comparisons, MNI coordinate: 33, -36, 34). **(C)** The white fiber tracts with higher RD values (*p* < 0.006, corrected for multiple comparisons, MNI coordinate: 32, -38, 36). Background images: standard MNI_T1_1mm template and the mean FA skeleton (Green). Warm colors (Red–Yellow): the corresponding value increases in the amusia group. Clusters of significant voxels are projected on the background images using the tbss_fill script in FSL to make the visualization easy.

**Table 2** shows abnormal white fiber tracts located by cluster peak information in the amusia group. No significant difference of FA was found between the two groups, while the other three diffusivity indices (AD, MD, and RD) were all higher in the amusics. Compared with the controls, amusics showed higher AD values in the corpus callosum (CC), the right inferior longitudinal fasciculus (ILF), and the right inferior frontal-occipital fasciculus (IFOF) (**Figure 1A**). Significant increases in both MD and RD were located in the right superior longitudinal fasciculus (SLF) (**Figures 1B,C**).

**Table 2:** Abnormal white fibre tracts located by cluster peak information in the amusia group assessed by diffusivity indices.

			Peak Information					
			Tract	MNI coordinate			*p*-value	
	Type	Cluster size		*X*	*Y*	*Z*		Overlap size with AF
**AD**								
	Increase	22	ILF.R1	36	-58	6	<0.001	5
	Increase	54	IFOF.R1	34	-47	10	<0.001	37
	Increase	214	IFOF.R2	29	-48	19	<0.001	8
	Increase	470	ILF.R2	39	-44	-7	<0.001	294
	Increase	732	CC	1	-28	22	<0.001	0
**MD**								
	Increase	32	SLF.R	33	-36	34	<0.001	17
**RD**								
	Increase	1060	SLF.R	32	-38	36	<0.006	154

*Significance was set at *p* < 0.001 for AD/MD and at *p* < 0.006 for RD (corrected for multiple comparisons). ILF, inferior longitudinal fasciculus; IFOF, inferior fronto-occipital fasciculus; SLF, superior longitudinal fasciculus; AF, arcuate fasciculus.*

In addition, in order to test whether there were any changes in the AF of amusics, we calculated the overlap area between the abnormal clusters obtained by TBSS and the AF template^3^. Four of the five abnormal clusters with higher AD overlapped with the right AF while the right SLF with higher MD/RD also contained part of the right AF (see the last column in **Table 2**).

#### Strength of Structural Connections

**FIGURE 2:**
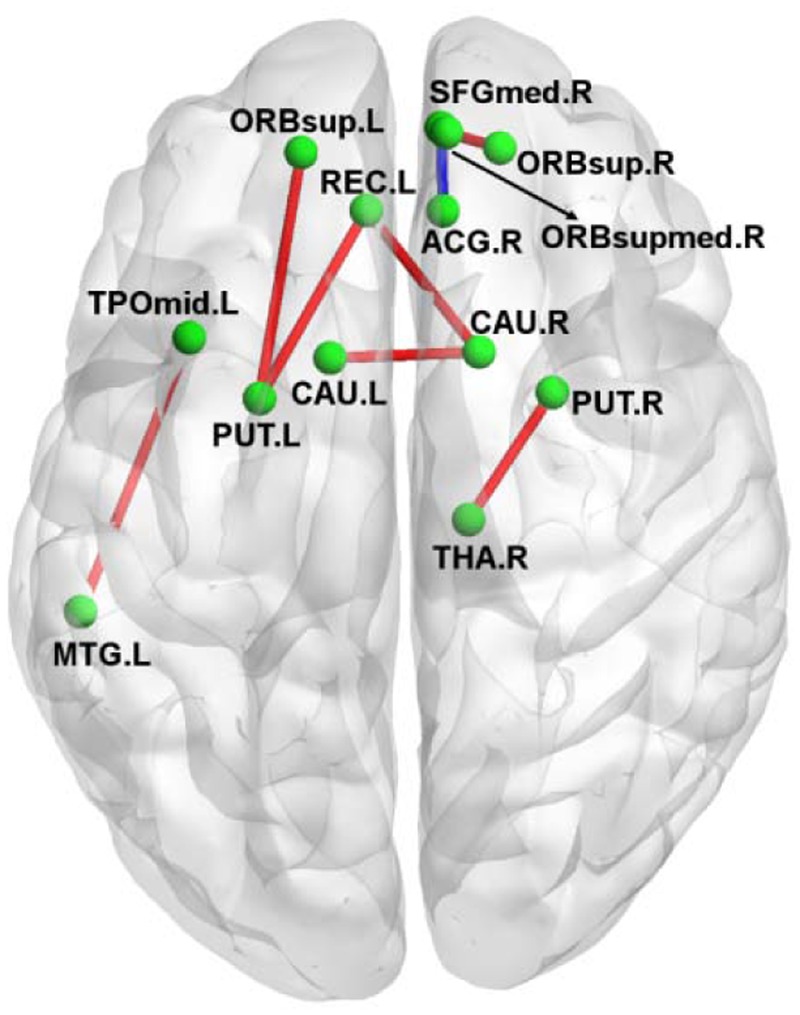
A 3D view of the difference in structural connections between normal controls and amusics. Red, stronger connections (more number of white fibers) in the amusia group. Blue, weaker connections (less number of white fibers) in the amusia group.

**Figure 2** shows the connection differences between the normal controls and the amusics after FWE correction (*p* < 0.05). Significantly weaker connection (the number of white fiber tracts was reduced) between the right anterior cingulum gyrus (ACG.R) and the right medial orbitofrontal cortex (ORBsupmed.R) was found in the amusics, while seven structural connections were stronger (the number of white fiber tracts increased) in the amusia group (see **Table 3**).

**Table 3:** The difference in structural connections (the number of WM fibers between each two nodes) between controls and amusics.

Type	Node 1	Node 2	Controls	Amusics
Amusics < Controls	ACG.R	ORBsupmed.R	32.8 (3.7)	5.4 (1.4)
	PUT.L	ORBsup.L	4.9 (1.4)	26.3 (7.4)
	SFGmed.R	ORBsup.R	0.2 (0.1)	1.0 (0.3)
	CAU.R	REC.L	2.0 (1.4)	15.2 (3.8)
Amusics > Controls	PUT.L	REC.L	1.2 (0.6)	10.2 (3.1)
	CAU.R	CAU.L	9.0 (4.2)	44.5 (9.4)
	THA.R	PUT.R	2.2 (1.0)	21.1 (5.4)
	TPOmid.L	MTG.L	7.6 (1.2)	17.8 (2.7)

*The abbreviations refer to Supplementary Table S1. *p* < 0.05, FWE correction.*

### Network-Based Analysis

**FIGURE 3:**
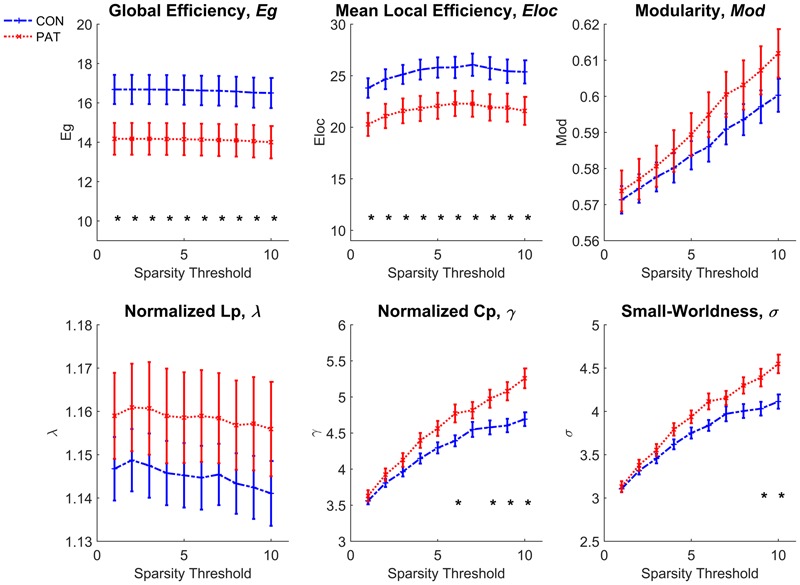
Comparison of global network properties between normal controls and amusics. CON, normal controls; PAT, amusics. ^∗^Significant difference between normal controls and amusics.

Comparison of the global topological measurements of WM networks is shown in **Figure 3**. The amusics exhibited both lower global efficiency and lower mean local efficiency across network thresholds 1 to 10 than normal controls. No statistical differences were found between the network modularity of the two groups. As for the comparison of small-worldness-related properties, the brain networks of both groups preserved small-worldness characteristics (σ ≫ 1, γ ≫ 1, and λ ≈ 1). But the amusia group had higher σ at thresholds 9–10 (bottom right of **Figure 3**) and higher γ at thresholds 6 and 8–10 (bottom center of **Figure 3**).

### Relationship between Tract-Based and Network-Based Analysis

**FIGURE 4:**
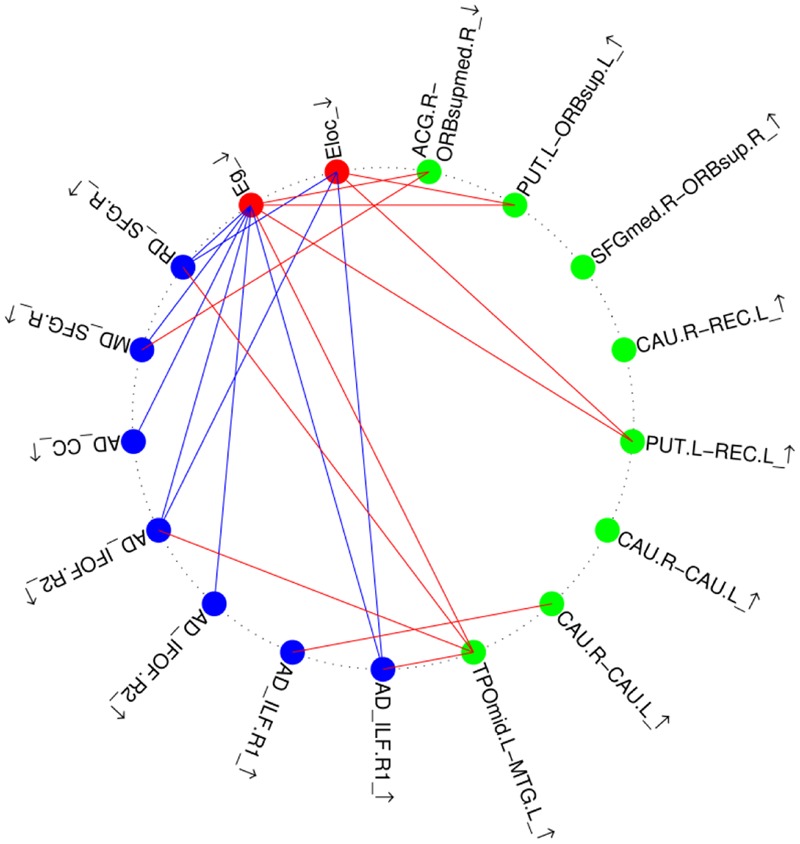
Relationship between altered brain morphological measures in the amusia group. Red nodes, altered global topological properties (see **Figure 3**); blue nodes, altered diffusivity indices (see **Table 2**); green nodes, altered strength of structural connections (see **Table 3**, A–B_arrow means the strength of structural connections between region A and region B, the abbreviations refer to Supplementary Table S1). ↑, Enlarged measures in the amusia group. ↓, reduced measures in the amusia group. Red lines, positive correlations between two measures; blue lines, negative correlations between two measures.

We calculated the correlation coefficients between the abnormal indices of two kinds of analysis in two groups. The results are shown in **Figure 4**. Only the RD value of the right SLF was negatively correlated with the global/mean local efficiency of the brain network in both the control and amusia groups. The other significant correlations between different indices were found only in the amusia group, not in the control group. Reduced network efficiency was negatively correlated with the majority of higher diffusivity indices and was positively correlated with the weaker connection (ACG.R-ORBsupmed.R) in the amusia group. Three stronger connections were positively correlated with reduced network efficiency. The correlation coefficients and the corresponding *p*-values are shown in Supplementary Table S3.

### Relationship with Musical Performance

In the control group, none of the abnormal indices had significant correlations with the musical scores. In the amusia group, we found that only a higher AD value of the right inferior frontal-occipital fasciculus was negatively correlated with the average musical score (*r* = -0.739, *p* = 0.006).

## Discussion

In the study, we investigated whole-brain changes of WM fibers in individuals with congenital amusia at both the local level (tract-based analysis) and the global level (network-based analysis). The main findings are that in the amusics: (1) increases of AD were located in the CC, the right IFOF, and the right ILF, while increases of MD and RD were both located in the right SLF; (2) the efficiency of the brain network was reduced in congenital amusia; (3) the reduced network efficiency was negatively correlated with the majority of higher diffusivity indices; (4) the enlarged AD value of the right IFOF was negatively correlated with musical scores. These results are consistent with our hypothesis that congenital amusia is a disconnection syndrome, which we discuss below.

Tract-based analysis of diffusivity indices showed no significant difference of FA between the controls and the amusics. FA is usually sensitive to WM microstructures, but is not sufficient to characterize the tissue changes alone (Alexander et al., 2007), because lower FA may be caused by lower AD or higher RD, and vice versa. More importantly, if AD and RD values both increase, the FA value may not change significantly. The other three diffusivity indices, AD, MD, and RD, were all found to be higher in the amusia group. Increases of MD and AD usually result from reduced cell density while increases of RD are usually related to impaired myelin (Alexander et al., 2007). The higher diffusivity indices indicate microstructural impairments of WM tracts in amusics.

When compared with the controls, amusics exhibited higher MD and RD values in the right SLF which, according to the overlapping analysis, contained part of the right AF. This result confirms previous findings that the fronto-temporal pathway, as a part of SLF, is abnormal in amusics (Hyde et al., 2011; Albouy et al., 2013; Lense et al., 2014). Considering the debate on the method of AF reconstruction in two previous studies (Loui et al., 2009; Chen et al., 2015), we did not conduct a direct comparison of AF volumes between the two groups. Nevertheless, our result that the abnormal cluster with higher MD/RD contained part of the right AF indicates the abnormality of the right AF in amusics. Loui et al. (2009) failed to track the right AF, and reported a reduced right AF volume in amusics. However, these results were not replicated in Chen et al.’s (2015) study. Considering that amusics rarely show other neurological symptoms, it seems highly unlikely that there is a complete absence of AF in amusics. Although the change of AF volume was not found by Chen et al. (2015), previous studies (Hyde et al., 2011; Albouy et al., 2013; Lense et al., 2014) and our own results are consistent with the suggestion that the fronto-temporal pathway is impaired in amusics. We infer that the AF structure may be altered (e.g., higher MD/RD), but it is unlikely that the whole AF structure will disappear (Loui et al., 2009).

Besides the abnormal SLF revealed by MD and RD, other WM tracts also exhibited abnormal AD. Higher AD was located in the CC, the right ILF, and the right IFOF. Higher AD usually results from reduced cell density (Alexander et al., 2007). This shows that there is abnormality not only in the fronto-temporal pathway, but also in other structural connections of the brain. The CC is the bridge between the left and right cortical hemispheres, and thus supports interhemispheric communication. An altered CC would likely affect the efficiency of information transfer between the two hemispheres in amusics.

Two clusters in the right ILF and two clusters in the right IFOF were also found to be changed in the amusics. Here, we should note again that the JHU–ICBM-DTI-81 white-matter tractography atlas used in the TBSS analysis is probabilistic. Some regions in this atlas are labeled as multiple WM tracts with relatively high probability. In our study, the two abnormal clusters labeled with relatively high probability in the right ILF (one with 53%, the other with 34%) are also in the right IFOF (one with 42%, the other with 24%). Previous studies have revealed spatial overlap between the ILF and IFOF (Ashtari, 2012; Pyles et al., 2013). These two abnormal clusters are therefore likely to be located in the overlapping region between the right ILF and IFOF. The abnormal clusters of the right ILF and the right IFOF are all located in the temporal lobe. Some musical information processed in the auditory cortex may be lost during the transfer to other parts of the cortex due to the impaired ILF and IFOF in amusics, which could lead to problems in distinguishing small differences in musical tone or recognizing familiar melodies in amusics. More importantly, the higher AD value of the right IFOF was negatively correlated with musical scores. This result suggests that the right IFOF plays an important role in the understanding of congenital amusia.

Previous studies have reported that the integrity of the IFOF is associated with the verbal fluency (Bauer et al., 2015) and the verbal memory (McDonald et al., 2008). Logically speaking, the alterations of the IFOF in amusics suggest that the nature of amusia might be linked to the poor verbal memory or verbal fluency. We would like to emphasize that the amusics in our study have no problem in daily communication. Moreover, no previous studies reported that amusics have verbal memory or verbal fluency problems. Thus, in our initial experimental design, we did not conduct tests on the verbal fluency or verbal memory.

The altered structural connections are mainly located at the superior frontal gyrus, the anterior cingulate cortex, the striatum (putamen and caudate) and the thalamus. Previous study found that the superior frontal gyrus was activated by the unfamiliar music (Plailly et al., 2007). Generally speaking, amusics are unfamiliar with almost all kinds of music (actually most of the tones are more or less the same for them; if they don’t know the lyrics, they can not recognize even those songs that they have well known before). We conjecture that the superior frontal gyrus of amusics is often activated, leading the connections of the interior superior frontal gyrus to be increased. The anterior cingulate cortex is involved with emotion information and processing (Etkin et al., 2011). The connections between the anterior cingulate cortex and the medial orbitofrontal cortex are decreased, which is consistent with the phenomena that if without knowing the lyrics, amusics cannot understand the emotions expressed in music. The striatum receives inputs from the thalamus and project principally to frontal areas (Herrero et al., 2002). As a locus of internal beat generation, the striatum responds more to beat rhythms than non-beat rhythms (Grahn and Rowe, 2009). The ascending projection systems from the thalamus convey tonal content and modulate cortical rhythmic context (Musacchia et al., 2014). That the regions within the striatum and thalamus are more connected may be caused by the compensatory mechanisms of the brain to restore the function of processing pitch/rhythm/melody.

The result that the efficiency of the WM network was reduced in congenital amusics is consistent with the previous study (Zhao et al., 2016). Global efficiency reflects the efficiency of information transfer between long-range connected regions while local efficiency reflects that between neighboring regions. This indicates that the efficiency of information transmission is reduced between both long-range and local regions in amusics. This result is also consistent with the abnormal WM tracts revealed by the tract-based analysis. We note that small-worldness was higher in the amusics, which was reflected by the significantly higher normalized clustering coefficient γ. The increased γ implies that the clustering coefficient of the amusics’ network is higher than that in an equivalent network with a random configuration, which suggests that amusics’ network shifts toward a network with a regular configuration.

White matter tracts (i.e., the bundles of myelinated axons) connect GM regions (i.e., the locations of nerve cell bodies) of the brain to each other, and carry nerve impulses between neurons. This means that WM tracts are the foundation of transmitting information in the brain network. The WM network is directly constructed from WM tracts. If one WM tract is abnormal, then the speed it transmits the neural signal will decrease, and in the extreme case it will be unable to transmit the neural signal. If only several bundles of WM tracts are abnormal, neural signals can be still transmitted via the loops constituted by other WM tracts, and the efficiency of brain network communication will not significantly alter. However, if a large number of WM tracts or those WM tracts that cannot be replaceable are abnormal, then the neural signals in the brain cannot be efficiently transmitted, which may make the network efficiency reduced. The TBSS analysis shows that the SLF, ILF, IFOF, and CC of amusics are all altered, which are highly likely to degrade the network efficiency of amusics. In the amusia group, reduced efficiency was negatively correlated with the higher diffusivity indices of impaired tracts detected by TBSS, and positively correlated with the weaker connection between the right ACG and the right ORBsupmed. This result suggests that altered tracts are strongly linked to reduced efficiency of the brain network. Therefore we conjecture that abnormal WM tracts lead to the reduced network efficiency. Furthermore, we found that three of the seven stronger structural connections (indexed by higher numbers of white fiber tracts) were positively correlated with the reduced efficiency of the brain network in the amusia group. The stronger the connections, the greater the network’s efficiency. Although network efficiency is lower in the amusia group, these stronger connections may be an attempt to restore network efficiency to the normal state. The larger number of WM fibers is likely a compensation for the impairments in amusics’ brains.

It may be doubted that the sample size is a little small to perform the network analysis. In the network analysis, we (1) investigated the alterations of six global topological measures across 10 thresholds (including global efficiency, mean local efficiency, modularity, and small-worldness quantified by normalized clustering coefficient over normalized characteristic path length) in the amusics and (2) directly compared the structure connections (i.e., the edge weight in the network, 90^∗^(90-1)/2 = 4005 edges) between two groups. For the former, we had adopted the FDR correction, suggesting that the expected statistical results of the Type I error rate should be no more than 0.05; while for the latter, we had adopted the stricter FWE correction, which means that the statistical results of the Type I error rate will be strictly no more than 0.05. In one word, our statistical analysis on Type I error conforms to standard statistical norms. Type II error refers to the probability that a measurement is abnormal yet has not been discovered. Due to the limited number of our samples, it is likely that some alterations in some measures cannot be detected; however, we would like to emphasize that the abnormal measures reported in this study are statistically significant.

Several points should be taken into consideration when interpreting the results. First, the number of participants was relatively small. Second, all participants were native speakers of Cantonese. The reduced efficiency of the WM network in amusics was both found in our study and Zhao’s study (Zhao et al., 2016). But the participants in these two studies are all native speakers of tonal languages (Cantonese in our study and Mandarin in Zhao’s study). Future studies are needed to replicate the current study and examine whether the current findings are generalizable to different language populations. Third, a relatively less stringent significant threshold (uncorrected *p* < 0.05) was used to build the bridge between different kinds of brain measures (local measures and global measures). The experimental results showed that the reduced network efficiency (global measures) was negatively correlated with the majority of higher diffusivity indices (local measures) in the amusia group. If a more stringent threshold, such as *p* < 0.001, was used, few significant correlations were detected. But we would like to emphasize that the negative correlation between higher diffusivity indices and reduced network efficiency (uncorrected *p* < 0.05) shows that these different kinds of brain indices have the tendency to be correlated. We infer the results that survivals can be detected for the uncorrected *p* < 0.05 while few survivals are detected for the corrected situation or *p* < 0.001 are possibly due to the relatively small amuisa samples in our study. The situation can be improved if more participants are recruited, which are actually one of the topics in our future research. Considering that amusia may be a genetic-related disease (Peretz et al., 2007), we conjecture that the genes related to WM fibers may be atypical. The atypical genes lead to impaired WM fibers (reduced efficiency of the brain network), which then leads to amusia in many individuals. This conjecture should be investigated by examination of the genes related to WM fibers.

## Conclusion

The current study combined tract-based and network-based analysis to investigate changes in WM tracts in congenital amusia. The impairments of WM tracts identified by tract-based analysis, including WM tracts with higher diffusivity indices and fewer WM tracts, were correlated with the reduced efficiency of the brain network in amusics. This suggests that impaired WM tracts lead to the reduced network efficiency. Our findings are consistent with the hypothesis that congenital amusia is a disconnection syndrome.

## Ethics Statement

This study was carried out in accordance with the recommendations of ‘Ethics Committee of the Chinese University of Hong Kong and the Institutional Review Board of the Shenzhen Institutes of Advanced Technology’ with written informed consent from all subjects. All subjects gave written informed consent in accordance with the Declaration of Helsinki. The protocol was approved by the ‘Ethics Committee of the Chinese University of Hong Kong and the Institutional Review Board of the Shenzhen Institutes of Advanced Technology.’

## Author Contributions

JW, CZ, SW, and GP designed the work. JW, CZ, SW, and GP wrote the article. JW, CZ, SW, and GP involved in the revision of the article. JW and SW contributed to the data analysis and statistical expertise. CZ and GP involved in data collection. CZ and GP involved in obtaining funds.

## Conflict of Interest Statement

The authors declare that the research was conducted in the absence of any commercial or financial relationships that could be construed as a potential conflict of interest.

## Footnotes

1 http://www.fmrib.ox.ac.uk/fsl

2 http://www.nitrc.org/projects/gretna/

3 http://www.natbrainlab.com/
