# Supplementary material for: Is Congenital Amusia a Disconnection Syndrome? A Study Combining Tract- and Network-Based Analysis
Source: Front Hum Neurosci. 2017 Sep 29;11:473. doi: 10.3389/fnhum.2017.00473 (PMC5626874; doi:10.3389/fnhum.2017.00473)
Supplement: Supplementary file 1 [file Data_Sheet_1.pdf]

Is Congenital Amusia a Disconnection Syndrome? A Study Combining Tract- and Network-Based Analysis

Jieqiong Wang, Caicai Zhang, Shibiao Wan, Gang Peng

Supplementary materials

Tables

Table S1 Brain regions defined in the AAL atlas in each hemisphere.

Abbreviation	Region	Abbreviation	Region
PreCG	Precentral gyrus	LING	Lingual gyrus
SFGdor	Superior frontal gyrus, dorsolateral part	SOG	Superior occipital gyrus
ORBsup	Superior frontal gyrus, orbital part	MOG	Middle occipital gyrus
MFG	Middle frontal gyrus	IOG	Inferior occipital gyrus
ORBmid	Middle frontal gyrus, orbital part	FFG	Fusiform gyrus
IFGoperc	Inferior frontal gyrus, opercular part	PoCG	Postcentral gyrus
IFGtriang	Inferior frontal gyrus, triangular part	SPG	Superior parietal gyrus
ORBinf	Inferior frontal gyrus, orbital part	IPL	Inferior parietal gyrus

ROL	Rolandic operculum	SMG	Supramarginal gyrus
SMA	Supplementary motor area	ANG	Angular gyrus
OLF	Olfactory cortex	PCUN	Precuneus
SFGmed	Medial superior frontal gyrus	PCL	Paracentral lobule
ORBsupmed	Medial orbitofrontal cortex	CAU	Caudate
REC	Gyrus rectus	PUT	Putamen
INS	Insula	PAL	Pallidum
ACG	Anterior cingulum gyrus	THA	Thalamus
MCG	Middle cingulum gyrus	HES	Heschl’s gyrus
PCG	Posterior cingulum gyrus	STG	Superior temporal gyrus
HIP	Hippocampus	TPOsup	Superior temporal pole
PHG	Parahippocampal gyrus	MTG	Middle temporal gyrus
AMYG	Amygdala	TPOmid	Middle temporal pole
CAL	Calcarine sulcus	ITG	Inferior temporal gyrus
CUN	Cuneus		

Table S2 Topological network properties used in the study.

**Global topological properties**	
Global Efficiency *Eg*	Mean value of all regions' nodal efficiency in the network, which reflects the efficiency of transferring information in the network
Mean Local Efficiency *Eloc*	Mean value of global efficiency of the sub-network constructed by the regions connected with the region *i*, which reflects the network ability to stay robust to attack
Modularity *Mod*	The level of dividing the network to distinct groups of nodes with denser connections within these nodes than with the others in the network.
Small-worldness σ	The small-world network is a type of network with a large clustering coefficient C_p and a small average shortest path length L_p. It reflects an optimal balance between functional integration and functional segregation

Table S3 Relationship between altered brain morphological measures in the amusia group.

			**Altered diffusivity indices**							**Altered structural connections**							
			AD_ILF.R1_↑	AD_IFOF.R1_↑	AD_IFOF.R2_↑	AD_ILF.R2_↑	AD_CC_↑	MD_SLF.R_↑	RD_SLF.R_↑	ACG.R - ORBsupmed.R_↓	PUT.L - ORBsup.L_↑	SFGmed.R - ORBsup.R_↑	CAU.R - REC.L_↑	PUT.L - REC.L_↑	CAU.R - CAU.L_↑	THA.R - PUT.R_↑	TPOmid.L - MTG.L_↑
**Altered global topological properties**	Eg_↓	*r*	-.705	-.324	-.586	-.698	-.612	-.588	-.614	.616	.847	.075	-.129	.730	-.343	-.353	.618
		p	**.010**	.304	**.045**	**.012**	**.035**	**.044**	**.034**	**.033**	**.001**	.818	.689	**.007**	.276	.261	**.032**
	Eloc_↓	*r*	-.601	-.189	-.551	-.719	-.514	-.482	-.612	.564	.840	.098	-.248	.714	-.298	-.281	.535
		p	**.039**	.557	.064	**.008**	.087	.113	**.035**	.056	**.001**	.763	.437	**.009**	.347	.376	.073
**Altered structural connections**	ACG.R - ORBsupmed.R_↓	*r*	-.350	-.282	-.249	-.505	-.347	-.648	-.428								
		p	.264	.375	.436	.094	.269	**.023**	.166								
	PUT.L - ORBsup.L_↑	*r*	-.555	-.331	-.477	-.572	-.465	-.520	-.553								
		p	.061	.293	.117	.052	.127	.083	.062								
	SFGmed.R - ORBsup.R_↑	*r*	.119	-.233	.010	.120	.017	-.157	-.078								
		p	.713	.466	.977	.711	.959	.626	.809								
	CAU.R - REC.L_↑	*r*	.128	.078	.056	.506	.064	.116	.478								
		p	.692	.809	.862	.093	.844	.720	.116								
	PUT.L - REC.L_↑	*r*	-.539	-.569	-.489	-.494	-.451	-.564	-.480								
		p	.071	.053	.106	.102	.141	.056	.114								
	CAU.R - CAU.L_↑	*r*	.532	.460	-.241	.302	.198	.284	.074								
		p	.075	.133	.451	.340	.538	.371	.818								
	THA.R - PUT.R_↑	*r*	.479	.643	.022	.281	.431	.557	.027								
		p	.115	**.024**	.945	.376	.162	.060	.934								
	TPOmid.L - MTG.L_↑	*r*	-.834	-.413	-.197	-.723	-.551	-.547	-.619								
		p	**.001**	.183	.540	**.008**	.063	.066	**.032**								

Note: significance was set at $p<0.05$

Figures

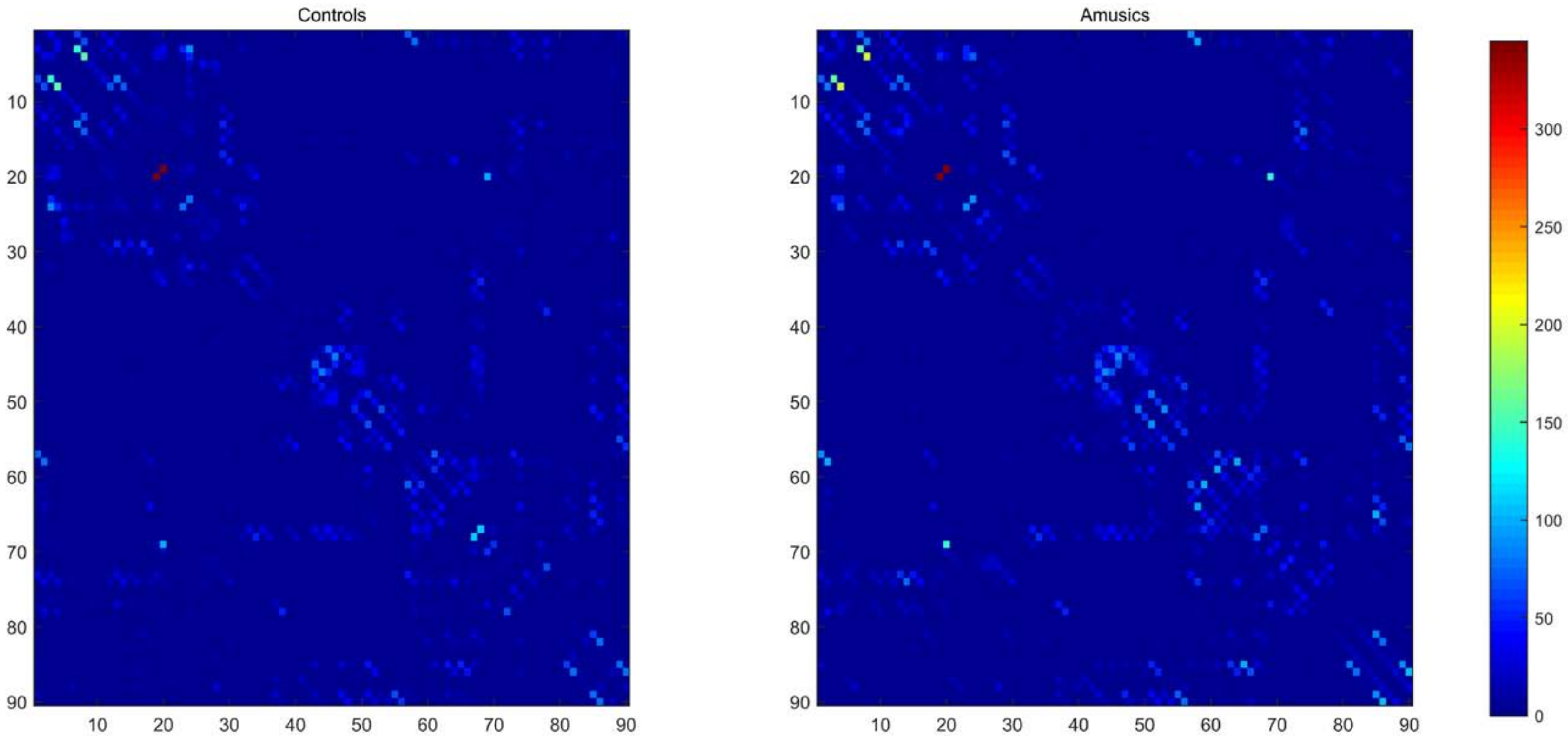

Figure S1 The connection matrices (at the threshold of 5) for 90 AAL regions of two groups.
